# Single nucleotide polymorphism of NLRP3 (Q705K) In juvenile spondyloarthritis and oligo/ polyarticular juvenile idiopathic arthritis

**DOI:** 10.1186/1546-0096-12-S1-P31

**Published:** 2014-09-17

**Authors:** Marija Perica, Mandica Vidovic, Lovro Lamot, Lana Tambic Bukovac, Sanja Kapitanović, Miroslav Harjaček

**Affiliations:** 1 Pediatric and Adolescent Rheumatology, Children's Hospital Srebrnjak, Croatia; 2 University of Zagreb School of Medicine, Zagreb, Croatia; 3 The Ruđer Bošković Institute, Zagreb, Croatia

## Introduction

The NLRP3 inflammasome is a key component of the innate immune system serving as an intracellular sensor of microbial components and cell injury. Gain-of-function mutations of the NLRP3 gene, such as single nucleotide polymorphism (SNP) Q705K, lead to autoproteolytic activation of caspase 1, resulting in excessive and uncontrolled production of proinflammatory cytokines. This may represent the mechanism by which an inflammatory loop is triggered leading to a long-term inflammatory phenotype.

## Objectives

Our objective was to compare the frequency of SNP Q705K of NLRP3 among patients with juvenile spondyloarthritis (jSpA) and juvenile idiopathic arthritis (JIA).

## Methods

DNA was extracted from blood samples of 37 jSpA patients and patients with oligoarticular or polyarticular JIA, diagnosed according to ILAR criteria. Polymorphism of the NLRP3 (Q705K) was determined using real time and multiplex PCR.

## Results

Among 37 genotyped patients, 24 patients with jSpA (92.31%) and 9 patients (81.82%) with JIA were carriers of the wild type allele. Only 2 patients in each group were heterozygous for NLRP3 (Q705K) polymorphism (7.69% in jSpA and 18.18% in JIA group). Although the observed frequency among groups was not statistically significant (Pearson Chi-square 0.8820724, p=0.64337), the frequency of allele polymorphism observed among our study population was higher (10.81%) than previously described in the general population (6.5%).

## Conclusion

The frequency of SNP Q705K of the NLRP3 gene did not differ among jSpA and JIA patients. There was also no evidence that variant of NLRP3 is a major risk factor for jSpA or JIA, however, lack of susceptibility should be confirmed in a larger group of patients.

## Disclosure of interest

None declared.

